# A manual three-step latent profile analysis to discover phubbing risk profiles among university students

**DOI:** 10.3389/fpsyg.2025.1510585

**Published:** 2025-06-25

**Authors:** Ömer Şimşek, Bülent Başaran

**Affiliations:** Department of Instructional Technologies, Dicle University, Diyarbakır, Türkiye

**Keywords:** phubbing, university students, latent profile analysis, manual three-step approach, distal outcomes, loneliness, smartphone

## Abstract

Phubbing, snubbing others in favor of one’s smartphone, is a growing concern among university students. This cross-sectional study aimed to identify distinct phubbing profiles among university students and examine the influence of various factors on these profiles, as well as their associations with loneliness, communication skills, and happiness. Latent profile analysis (LPA) was conducted on a convenience sample of 691 university students (71.9% female, 28.1% male; M = 22.50 years) from a state university in southeastern Turkey. The study found three unique phubbing profiles: Low (67.3%), Moderate (24%), and High (8.7%) phubbing risk. Gender and smartphone addiction were significant predictors of phubbing profiles, with females and those with higher smartphone addiction showing a higher likelihood of engaging in phubbing. Interestingly, insomnia, depression, socioeconomic status, number of friends, and frequency of social interactions did not significantly influence phubbing profiles. The study found that a higher risk of phubbing was associated with increased loneliness; however, no significant differences were observed between profiles in terms of communication skills or happiness. These findings underscore the importance of addressing smartphone addiction as a critical driver of phubbing and the potential for phubbing to exacerbate loneliness among university students. The study also suggests that future research should delve into the long-term effects of phubbing on social and psychological well-being and develop interventions targeting healthy digital behaviors.

## 1 Introduction

In this digital age, the widespread use of smartphones has profoundly altered social interactions, prompting and facilitating new phenomena such as ‘phubbing, ‘which is defined as ‘phone snubbing’ (David and Roberts, 2017; Chotpitayasunondh and Douglas, 2018). Phubbing refers to the tendency to ignore people in a social context by looking at and using a smartphone. It indicates a subtle social and behavioral disorder encompassing various forms of internet, social media, and gaming addictions, as well as those related to smartphone use, distinguishing it from other mobile phones (Ivanova et al., 2020; Kwon et al., 2013; Zhao et al., 2021). This social and behavioral disorder is becoming increasingly prevalent among younger generations, including university students (Ríos Ariza et al., 2021; Barbed-Castrejón, 2024; Roberts et al., 2014). The rising prevalence rates reported in numerous studies across various countries and student populations highlight that phubbing is a common phenomenon among university students and underscores its significance (Lai et al., 2022; Barbed-Castrejón, 2024; Şahin et al., 2024).

Phubbing behavior is rooted in the digital ecosystem and the backdrop created by technological developments (Altay Barak and Kabasakal, 2023; Karataş et al., 2024). Factors such as the tendency to respond to push notifications (Parus et al., 2021) and the fear of missing information (FOMO; Franchina et al., 2018) trigger this behavior. University students constitute a demographic group particularly vulnerable to phubbing due to their heavy smartphone use (Sun and Wong, 2024; Talan et al., 2023) and high interaction with social media platforms (Hunter-Brown, 2021). For these students, smartphones can function as a ‘safe base’ in regulating negative emotions (e.g., boredom, loneliness, anxiety), especially as a temporary relief from the challenges of university life such as stress, social anxiety, and loneliness, or as a means of compensating for perceived social deficiencies (Compensatory Internet Use Theory; Kardefelt-Winther, 2014; Karadağ et al., 2015; Türe Orhan, 2023; Yuchang et al., 2017; Schimmenti et al., 2013). In this context, the relationship that individuals establish with their phones can replace or interrupt traditional patterns of interpersonal attachment and social interaction (David and Roberts, 2017; Konok et al., 2016). Opportunities to be liked, appreciated, and to express oneself in a virtual environment (Altay Barak and Kabasakal, 2023) may contribute to the continuation of phubbing, especially among students with a lack of self-control (Türe Orhan, 2023) or technology-based addiction tendencies. The fact that smartphone use prevents face-to-face interactions reveals the potential for social ignoring, which can exacerbate feelings of social isolation and emotional dysregulation among students (Rachman et al., 2019; Han et al., 2022), ultimately negatively affecting their academic and social lives (Rachman et al., 2019; Güngör and Kurt, 2024).

Phubbing significantly reduces the quality of communication, as it introduces distractions during face-to-face interactions due to smartphone usage (Parmaksız, 2021; Ayar and Gürkan, 2021). Behaviors like checking social media or responding to messages reduce trust in relationships while fostering negative emotions such as conflict or depression (Roberts et al., 2014; Chotpitayasunondh and Douglas, 2018). It also decreases life satisfaction and relationship quality by creating emotional distance and weakening shared experiences in friendships (Sun and Samp, 2021). However, some studies have reported that students exhibiting high levels of phubbing behavior are happier (Alizamar et al., 2019). Compensatory internet use theory (Kardefelt-Winther, 2014) proposes that individuals may turn to the internet to fill unmet offline needs. In this context, excessive smartphone use can serve as a substitute for feelings of boredom or loneliness (Al‐Saggaff and O’Donnell, 2019). Consequently, the emotional states of individuals who phub and those who are phubbed may diverge regarding life satisfaction. For instance, phubbing, as a form of social exclusion, causes individuals to feel neglected and worthless (Williams, 2007), thereby reinforcing feelings of loneliness and adversely affecting personal well-being (Chotpitayasunondh and Douglas, 2018; David and Roberts, 2017; Wang et al., 2017; Maftei and Măirean, 2023). Nonetheless, loneliness can function both as a cause and a consequence of phubbing (Ivanova et al., 2020; Yam and Kumcağız, 2023). In this regard, phubbing is a multifaceted phenomenon that can influence individuals’ social interactions, psychological states, and various behaviors, including life satisfaction, across a broad spectrum. This study will examine loneliness, happiness, and communication skills as long-term effects of phubbing behavior.

Research on phubbing behavior yields unclear results regarding how demographic factors like gender and age influence its occurrence. Studies indicate that women exhibit higher rates of phubbing, as they tend to use phones to maintain social relationships, according to Lai et al. (2022). The academic literature presents inconsistent findings; some research identifies higher phubbing rates in women and older adults (Bitar et al., 2022), while other studies show that male students engage in more phubbing behavior (Chi et al., 2022). The connection between socioeconomic status (SES) remains uncertain, as higher SES appears to encourage greater smartphone usage, which may lead to phubbing behaviors (Tekkam et al., 2020), although conclusive evidence is lacking. The phenomenon of phubbing among university students arises from various interacting factors, including personal traits, technological usage patterns, and social relationships.

Existing research provides detailed knowledge about phubbing-related behaviors, but most studies use correlational methods to explore general relationships. There is a lack of understanding regarding how different elements interact and how various phubbing profiles result in unique consequences. Research needs to move beyond basic assessments of whether phubbing exists in behavior to investigate the different levels of phubbing, along with their related antecedents and resulting outcomes. This study utilizes the Latent Profile Analysis (LPA) method to uncover latent subgroups that basic descriptive statistics fail to detect (Spurk et al., 2020; Wardenaar, 2024; Liao et al., 2023). The study employs a manual three-step LPA process to conduct in-depth analyses of theoretical and empirical links between complex response patterns (profiles) and distant outcomes. The analysis examines how profile patterns relate to distant outcomes such as loneliness, happiness, and communication skills among university students while accounting for classification uncertainty. The research explores the effects of antecedent variables including gender, smartphone addiction, insomnia, depression, number of friends, social interaction frequency, and socioeconomic status on the phubbing profiles observed in university students. Furthermore, the study evaluates how these identified profiles correlate with crucial distal outcomes, including loneliness, happiness, and communication skills, which impact both mental health and academic success. Through our analysis of different profiles, we aim to provide essential information for developing specific intervention programs and support systems that cater to diverse student groups to safeguard social and psychological well-being.

## 2 Literature review

In this section, we review the literature on the prevalence of phubbing behavior among university students, its emergence, and the distal consequences that this behavior can trigger. However, the variables we use do not capture the full complexity of phubbing behavior. We believe that examining phubbing behavior across various profiles with a wide range of variables can provide nuanced contributions to the literature.

### 2.1 Phubbing prevalence among university students

Phubbing behavior among university students has emerged as a significant global issue, with prevalence rates varying across countries, generally ranging from moderate to high levels. Recent research underscores the widespread nature of this phenomenon and highlights potential contributing factors. For instance, a study conducted in Spain reported that half of university students exhibited phubbing behaviors, a rate notably higher than that observed among younger student groups (Barbed-Castrejón, 2024). Similarly, Şahin et al. (2024) investigated 869 undergraduate students in Turkey and found a prevalence of 7.9%, highlighting that female students, those from higher socioeconomic backgrounds, and individuals who spent more than 6 h daily on smartphones had a higher tendency toward phubbing. Supporting these findings, another study from Turkey revealed a moderate positive relationship between experiencing phubbing and engaging in the behavior itself, suggesting that exposure increases the likelihood of adopting such behaviors (Sönmez Sarı et al., 2023). Additionally, Ahmed et al. (2023) reported that 88.8% of international students in Turkey engaged in phubbing.

Comparable prevalence rates have also been identified in various cultural contexts. For example, research in India revealed phubbing prevalence rates ranging from 42.7% among medical students (Purwar et al., 2023) to 49.3% in broader university student samples (Davey et al., 2018). Consistently, a study by Tekkam et al. (2020) involving youth in Hyderabad also found a high prevalence of 52%. Similar findings were reported in a study conducted in the United States, where pharmacy students had a prevalence rate of 41.3% (Lo et al., 2022). In Indonesia, approximately 45.2% of university students exhibited high phubbing behaviors, influenced by factors such as smartphone addiction, internet addiction, social media dependency, and gaming addiction (Alizamar et al., 2019). Additional studies have explored phubbing among nursing students in South Korea (Han et al., 2022) and among university students in Peru, where regular phubbing was linked to disrupted sleep patterns and increased interpersonal conflicts (Ríos Ariza et al., 2021).

Overall, these studies suggest that phubbing among university students is a global challenge with varying prevalence rates. These variations result from differences in measurement tools, sample characteristics, cultural contexts, and specific social conditions present during data collection, such as during or after the COVID-19 pandemic.

### 2.2 The effect of demographic factors on phubbing

Based on empirical findings, the effects of the variables overshadow the associations between phubbing behavior and age and gender. Researchers have noted that the influence of gender on phubbing also reveals mixed reactions. Some studies indicate that females and older demographic groups are significantly more likely to engage in phubbing behavior than males (Bitar et al., 2022). However, other research has shown that boys are more susceptible than girls (Chi et al., 2022; Peleg and Boniel-Nissim, 2024). In one study, Cebollero-Salinas et al. (2022) found that females reported slightly higher levels of phubbing than males. Conversely, Etchezahar et al. (2023) indicated that phubbing exhibited no statistically significant gender-related relationships. Such contradictions in findings suggest that the relationship between phubbing and ethnicity can be inconsistent, depending on specific cultural contexts and demographic groups (Barbed-Castrejón, 2024; Masrukhin, 2023). Therefore, there are no significant gender differences in phubbing, and the existing literature on gender is not comprehensive (Thabassum, 2021).

In light of current empirical studies, phubbers aged 18 to 29 are more prevalent than those from other generations. For instance, Bitar et al. (2022) noted that younger users are more prone to phubbing. Similarly, Maftei and Măirean (2023) pointed out that the frequency of phubbing has decreased. As age has increased, so too have the observations of disapproval regarding the activity of phubbing. Moreover, Maftei and Măirean (2023) observed that younger users are more likely to use their mobile phones compared to 18-year-olds, especially university students. This observation explains why more phubbing behavior is noted among 18-year-olds. Peleg and Boniel-Nissim (2024) suggest an important finding: younger university students also tend to be heavier users and owners of multiple mobile phones. Similarly, Şahin et al. (2024) reported that most phubbers were university students across all human mobility regimes.

The relationship between socioeconomic status (SES) and phubbing yields mixed results. Some studies link higher SES to increased phubbing (Tekkam et al., 2020). Conversely, others associate lower SES indicators like crowding with less phubbing (Bitar et al., 2022) or find no correlation with income (Şahin et al., 2024). SES might also moderate phubbing’s effects, intensifying social anxiety under financial hardship (Chu et al., 2021), suggesting a complex, context-dependent association.

As demonstrated by the distinction in interpersonal friendship, ‘phubbing’ from smartphones is an essential topic of investigation. Miller-Ott et al. (2012) indicated that commercial friends were more likely to use their cell phones in various social situations than those with perceived self-orientation. They also indicated that cellphone use could lead to reduced satisfaction within friendships and cause more feelings of social loneliness. Phubbing can have negative ramifications on the quality of attachment in friendships.

The literature suggests that several antecedents of phubbing behavior exist among college students, and that this behavior has significant harmful effects. In this context, Table 1 summarizes the leading causes and associated negative consequences of phubbing among university students.

**Table 1:** Antecedents of phubbing among university students and associated harmful effects.

Antecedent	Associated harmful effects (with supporting identities)
Smartphone Addiction	Increased loneliness, reduced face-to-face communication (Ivanova et al., 2020; Chu et al., 2021)
Fear of Missing Out (FOMO)	Higher anxiety, social comparison stress, and problematic smartphone use (Karadağ et al., 2015; Safdar Bajwa et al., 2023)
Social Comparison	Low Self-Esteem, Poor Academic Performance (Chi et al., 2022; Safdar Bajwa et al., 2023)
Empathy Deficiency	Reduced Empathy, Weakened Social Bonds (Uncu et al., 2024; Chotpitayasunondh and Douglas, 2018)
Sleep Problems	Poor sleep quality exacerbates smartphone overuse, emotional distress and increased Loneliness (Ríos Ariza et al., 2021; Tekkam et al., 2020)
Socioeconomic Status	higher status is associated with increased phubbing (Chu et al., 2021); TekkamHigher status is associated with increased phubbing (Tekkam et al., 2020) and(financial hardship) may exacerbate peer phubbing (Chu et al., 2021)
Gender Differences	Females often exhibit higher phubbing rates, potentially linked to social media engagement and relational needs; Bitar et al. (2022), Escalera-Chávez et al. (2020), Barbed-Castrejón (2024)
Communication Skill Deficits	Reduced interpersonal trust, lower communication quality, and diminished relational satisfaction (Ayar and Gürkan, 2021; Chotpitayasunondh and Douglas, 2018)
Depression and Anxiety	Elevated depressive symptoms, anxiety, and lower life satisfaction (Ivanova et al., 2020; Arshad, 2024)

Table 1 shows that there is a complex interplay between the antecedents of phubbing behavior and its harmful effects, and that cultural, demographic and interdisciplinary differences shape these dynamics. Accordingly, students’ tendency to use their devices excessively and uncontrollably is closely related to psychological problems such as loneliness and depression (Ivanova et al., 2020; Chu et al., 2021). University students’ increased focus on their phones and thus decreased face-to-face communication may contribute to feeling lonely and showing depressive symptoms (Ivanova et al., 2020). Therefore, smartphone addiction may significantly contribute to social isolation by causing young adults to prioritize their phones even in social settings (Karadağ et al., 2015). Again, university students’ anxiety about missing out on what is happening on social media or in the online world (FOMO) exacerbates feelings of anxiety and isolation by reinforcing the frequent checking of smartphones (Safdar Bajwa et al., 2023; Karadağ et al., 2015). In addition, students frequently engage in social comparison, especially through social media, which negatively impacts self-esteem and academic performance (Safdar Bajwa et al., 2023; Chi et al., 2022). A reduced sense of empathy also plays a critical role, increasing the likelihood of engaging in phubbing behaviors, further weakening interpersonal connections and overall empathy levels (Chotpitayasunondh and Douglas, 2018; Uncu et al., 2024). Sleep problems, which are common among university students, can both cause and contribute to phubbing; late-night phone use disrupts sleep patterns and increases feelings of loneliness (Ríos Ariza et al., 2021; Tekkam et al., 2020). Finally, socioeconomic status influences the extent and consequences of phubbing behaviors, while high SES is associated with more phubbing (Tekkam et al., 2020), low SES (financial hardship) may exacerbate the negative consequences of peer phubbing, such as social anxiety (Chu et al., 2021).

Overall, phubbing behavior is associated with many variables due to demographic factors, mobile addiction, frequency of social activities, and socioeconomic contexts. However, the existing research cannot explain in depth how these factors affect people at different levels of phubbing. Therefore, it is crucial to investigate phubbing and determine how it relates to demographic factors.

However, the main body of research suggests that phubbing potentially leads to increased loneliness, poorer communication skills, and poorer life satisfaction. It is also associated with psychological distress, depressive symptoms, anxiety and higher levels of stress in a sample of college students. As mentioned above, studies have shown that various variables, including gender, age, smartphone addiction, sleep disruptions, depressive traits, the number of social contacts, frequency of social interactions, and socioeconomic status influence the use of mobile devices. However, the current literature lacks systematic reporting of the actual outcomes or relationships between these factors and the use of phubbing at varying levels.

### 2.3 Distal outcomes of phubbing: loneliness, happiness and communication skills

#### 2.3.1 Loneliness

Loneliness can be defined as a subjective mismatch between an individual’s desired social connections and the quality of their existing social relationships (Seemann, 2022). In other words, loneliness is the feeling of being socially isolated or lacking sufficient or satisfying relationships (Perlman and Peplau, 1982). In this regard, loneliness possesses a subjective nature and does not solely imply being physically alone; a person may feel lonely even when in a social environment (Hawkley and Cacioppo, 2010). This phenomenon is increasingly recognized as a significant issue with far-reaching consequences for both individual and societal well-being (Bound Alberti, 2018; Safdar Bajwa et al., 2023). Theoretical studies on loneliness indicate that this feeling is linked to unmet basic human needs. The need to belong refers to people’s fundamental desire to have social relationships and to be accepted by others (Chotpitayasunondh and Douglas, 2018). When this need is not satisfied, loneliness may emerge. In this context, phubbing can violate essential human needs such as belonging (Chotpitayasunondh and Douglas, 2018).

According to Karadağ et al. (2015), higher education students prefer to use their smartphones more often when they feel lonely because they believe that doing so will alleviate their loneliness. Therefore, research shows that there is a significant relationship between phubbing behavior and loneliness (Sun and Wong, 2024; Yaseen et al., 2021). Since phubbing leads to perceived social exclusion by diminishing relational intimacy and satisfaction (Chotpitayasunondh and Douglas, 2018; Karadağ et al., 2015), university students report an increase in loneliness (Yam and Kumcağız, 2023; Büyükşakar and Çelik, 2024). Consequently, individuals experiencing loneliness are more likely to engage in phubbing as a means to escape feelings of isolation. As a result, phubbing can further isolate individuals and create a vicious cycle of loneliness and social disconnection (Sun and Wong, 2024). Accordingly, loneliness can function both as a cause and a consequence of phubbing (Ivanova et al., 2020; Yam and Kumcağız, 2023). Phubbing may contribute to heightened loneliness among university students during a critical period of social development and relationship building. Understanding this impact is essential for developing strategies to promote healthier communication habits and foster a greater sense of belonging within the university community.

#### 2.3.2 Happiness

Happiness, in general, can be defined as the state of emotional well-being experienced by individuals (Happiness, 2025), the general satisfaction with one’s life, evaluations of the quality of life, and the state of frequently feeling positive emotions (Savi Çakar and Karataş, 2017). This emotion can be distinguished from negative emotions such as sadness, fear and anger as well as other positive emotions such as love and excitement (Happiness, 2025). Social relationships, health, economic status, personal values and life events are important factors affecting the level of happiness (Alizamar et al., 2019). In this respect, happiness means a more permanent state of well-being and life satisfaction rather than just a momentary emotional state.

The relationship between phubbing behavior and various indicators such as well-being, life satisfaction, and mood-which are particularly important for university students-has been examined in numerous studies. Phubbing can threaten the basic need for belonging, which is intrinsically linked to happiness (Chotpitayasunondh and Douglas, 2018). For instance, research has demonstrated a negative correlation between phubbing and life satisfaction, as well as a positive correlation with depression (Barbed-Castrejón, 2024). A study involving Indonesian students indicated that those who exhibit high levels of phubbing are not necessarily unhappier, suggesting that while phubbing may hinder overall happiness, it does not directly affect individual happiness in daily life (Alizamar et al., 2019). Additionally, FOMO (Fear of Missing Out) related to phubbing has been shown to significantly impact well-being and partially mediate the relationship between phubbing and life satisfaction (Joshi, 2023). Supporting these findings, Aydin et al. (2024) defined two different profiles-Low Digital Addicts and High Digital Addicts-through Latent Profile Analysis (LPA) in their study with adult participants aged 18–65, examining the effects of digital obesity and phubbing behaviors on individuals’ well-being and social lives. This study found that phubbing behavior significantly negatively affected life satisfaction through relationship satisfaction, suggesting that phubbing indirectly diminishes individuals’ psychological well-being by weakening social connections. All this evidence strongly indicates the detrimental effect of phubbing on university students’ psychological well-being, highlighting a significant relationship with increased negative emotions and decreased life satisfaction. Although there is robust evidence regarding the negative effects of phubbing on university students’ psychological health, the importance of understanding the underlying mechanisms and potential interventions among different phubbing groups is emphasized (Altay Barak and Kabasakal, 2023).

#### 2.3.3 Communication skills

Communication skills encompass individuals’ ability to effectively convey their thoughts, feelings, knowledge, and needs through both verbal and non-verbal methods, as well as their capacity to accurately comprehend the messages of others (Ayar and Gürkan, 2021; Uncu et al., 2024). Effective communication consists of various components, including active listening, empathizing, speaking clearly and intelligibly, employing appropriate body language, and maintaining eye contact (Uncu et al., 2024). Strong communication skills play a fundamental role in establishing, maintaining, and developing healthy social relationships.

Studies indicate that phubbing behavior significantly negatively impacts university students’ communication skills. A study by Ayar and Gürkan (2021) involving 587 university nursing students found a moderate, significant, and negative correlation between smartphone addiction, phubbing behaviors, and communication skills. The study also revealed that students with high phubbing behaviors scored low in communication skills. In fact, phubbing behaviors were identified as a crucial factor (56%) influencing nursing students’ communication skills.

A qualitative study by Karadağ et al. (2015) reveals that young adults who exhibit phubbing behavior experience deficiencies in communication skills, have difficulty making eye contact during conversations, and misunderstand what is being said. Some participants stated that they completely disconnected from the social environment while using smartphones. Considering the importance of eye contact and careful listening in face-to-face communication, phubbing negatively affects these basic communication behaviors and reduces the quality of communication (Uncu et al., 2024).

Uncu et al. (2024) found a strong negative relationship between phubbing levels and empathic tendencies of university students. Empathy is an important part of effective communication and refers to the ability to understand and share the feelings of others. The fact that phubbing behavior decreases empathic tendencies may lead students to be less sensitive to the emotional needs of others while communicating and thus to communicate less effectively.

In another study by Han et al. (2022), a negative correlation was found between phubbing and interpersonal competence. Interpersonal competence refers to the ability to succeed in social interactions and establish positive relationships. The fact that phubbing reduces interpersonal competence may make it difficult for students to establish effective and healthy communication with their social environment. The study also states that students with low communication skills exhibit more phubbing behaviors.

### 2.4 The present study

The present study aims to advance the field and identify meaningful patterns of phubbing behaviors through latent profile analysis. Latent Profile Analysis (LPA) is a powerful statistical technique for examining intrapersonal behaviors and characteristics. It can be applied in various research scenarios across the social sciences. This technique is instrumental in identifying or discovering latent groups or differences in a given data set that would otherwise have gone unnoticed. Specifically, a manual three-step LPA is used to examine the impact of heterogeneous combinations of variables that cannot be discerned using traditional correlational methods. The manual three-step latent profile analysis approach examines the relationships between latent profile membership and exogenous variables, taking classification uncertainty into account (Bondjers et al., 2018). Unlike standard one-step or two-step approaches, this method allows latent profiles to be initially identified and then their relationships with exogenous variables to be analyzed (Vermunt, 2010).

The research examines the associations between identified profiles and several outcomes, including loneliness, communication competencies, and happiness. Furthermore, predictors relevant to student phubbing profiles are also considered. The findings can help close research gaps in the existing literature and provide insights into the features, drivers, and implications of phubbing in a university setting.

Research questions

1. What is the typology of phubbing profiles among university students?
2. How do covariates such as gender, age, smartphone addiction, insomnia, depression, number of friends, frequency of meeting friends, and socio-economic status influence the phubbing profiles of students?
3. What associations exist between distal outcomes such as loneliness, communication skills, happiness, and the phubbing profiles of university students?

## 3 Methods

### 3.1 Research design

This investigation uses college students’ phubbing actions using survey methodology and advanced statistical analysis (LPA). This layout allows for data to be gathered simultaneously, which permits later comparisons and correlation analysis (Creswell and Creswell, 2018).

### 3.2 Study group

This design was carried out at a state university in the southeastern region of Turkey. The study included 691 participants, consisting of students from various fields specific to this area. The sample comprised 497 female (71.9%) and 194 male (28.1%) students. Participants’ ages ranged from 17 to 57 years, with a mean age of 22.50 years (SD = 4.829). The study employed a convenience sampling method. Participants were recruited through the university’s online educational platform, where an invitation to participate in the research was distributed to students across various departments. This approach was selected due to its practicality in accessing the university student population during the data collection period and its efficiency in recruiting an adequate sample size within the constraints of our research timeline and resources. In the online survey for students, consent was obtained using a checkbox at the beginning, and participants were informed that they could skip questions or leave the survey at any time without facing any consequences. Focusing on the sample from Turkey, our research aims to provide an essential regional perspective on this global issue. According to the 2022 PISA report, which provided the data, it was observed that Turkish young people, when they are separated from their digital devices, exhibit significant levels of anxiety, ranking third among 37 OECD nations.

### 3.3 Data collection

A cross-sectional approach was utilized in this study to explore the impact of various factors on university data collected in the 2022–2023 Autumn semester following the easing of COVID-19 restrictions. The survey was distributed to university students through notifications on the institution’s educational platform. It was conducted online from October 1, 2022, to December 20, 2022, to gather feedback from students affected by the pandemic. The survey included questions about demographics and brief statements such as “I lack companionship,” to be completed within 8–12 min, focusing mainly on Oxford Happiness and UCLA scales.

Participation in the study was voluntary, subject to students agreeing to a document that allowed them to withdraw at any time. This approach resulted in a total of 691 complete responses, providing a comprehensive dataset for analysis without any missing information.

#### 3.3.1 Phubbing scale

Various measurement tools have been developed to assess phubbing behaviors (Chotpitayasunondh and Douglas, 2018; David and Roberts, 2020). The Generic Phubbing Scale (GPS), developed by Chotpitayasunondh and Douglas (2018), is one such tool. The GPS consists of four subscales: (1) Nomophobia (anxiety about being separated from one’s phone), (2) Interpersonal Conflict (perceived conflict with others due to phone use), (3) Self-Isolation (avoiding social activities in favor of smartphone use), and (4) Problem Acknowledgment (recognition of a phubbing problem). This scale includes 15 items divided into four factors, rated on a 7-point Likert scale ranging from 1 (Never) to 7 (Always). Scores for each factor are summed and averaged, with higher scores indicating stronger feelings related to the constructs of the respective subscales. In this study, the Cronbach’s alpha coefficient for internal consistency was found to be 0.87, indicating strong reliability.

#### 3.3.2 Communication skills scale

The Communication Skills Scale for Adults was developed by Korkut-Owen and Bugay (2014) to assess an individual’s communication skills. This Likert-type scale consists of 25 items, ranging from “always” to “never,” with scores ranging from a minimum of 5 to a maximum of 25. An individual’s score on the scale reflects their perceived competence in communication skills. The scale is divided into five factors: the first factor, Basic Skills and Self-Expression (BS-SE), consists of nine items; the second factor, Attention to Communication (AC), includes five items; the third factor, Willingness to Establish Relationships (WER), contains three items; the fourth factor, Effective Listening and Non-Verbal Communication (ELNVC), comprises five items; and the final factor, Adherence to Communication Principles (ACP), encompasses three items. The internal consistency reliability of the scale was evaluated using Cronbach’s alpha coefficient, yielding a value of 0.94. In this study, the alpha coefficient for internal consistency was determined to be 0.88.

#### 3.3.3 UCLA loneliness scale

The UCLA Loneliness Scale, developed by Hays and DiMatteo (1987), was adapted into Turkish for youth by Yıldız and Duy (2014). Three items were removed from the original scale based on confirmatory factor analysis results during the adaptation process. The adapted scale consists of seven items, including statements like “I have no friends” and “I feel left out.” It has a 4-point response range, with the fifth item reverse scored. Higher scores on the scale indicate a greater intensity of general loneliness. The confirmatory factor analysis demonstrated good model fit (χ^2^/df = 1.94, RMSEA = 0.06, AGFI = 0.95, GFI = 0.97, CFI = 0.998, and SRMR = 0.04). The Cronbach’s alpha coefficient for the scale’s internal consistency was reported as *α* = 0.74 in the adaptation study, while it was calculated as 0.69 in this study. To make the scale comparable to other distal outcomes (happiness [min = 1, max = 5] and communication skills [min = 1, max = 5]) min-max normalization was applied to loneliness scale as min = 1, max = 5. This method allows direct comparison by transforming all variables into a 1–5 range.

#### 3.3.4 Oxford happiness questionnaire short form

The OHQ-SF, initially conceptualized by Hills and Argyle in 2002 in English, constitutes a 7-item instrument to assess an individual’s degree of happiness. The Turkish adaptation of this measurement tool was executed by Doğan and Akıncı Çötok (2011). Participants concur with various statements using a 5-point Likert scale, where one denotes (Strongly Disagree) and five signifies (Strongly Agree). The Turkish adaptation demonstrated notable reliability, with internal consistency and test–retest reliability coefficients quantified at 0.74 and 0.85, respectively. This investigation revealed a Cronbach’s Alpha coefficient of 0.76.

### 3.4 Analytical strategy

This study used the R library ‘MplusAutomation’ to run the Mplus software in the R environment. This approach allows complex statistical analyses (especially LPA) to be performed in a more user-friendly and efficient way. MplusAutomation combines Mplus’s powerful modeling capabilities with the flexible data processing and visualization features of R, providing researchers with a comprehensive analysis environment.

LPA makes qualitatively different variable configurations salient that other analysis methods do not easily represent. This approach also helps understand the complex relationships between Phubbing, covariates, and distal outcomes. In this way, researchers can analyze complex social and psychological phenomena in more depth (Gezgin and Türk-Kurtça, 2023).

### 3.5 Stages of the analysis

*Step 1*, firstly, as there are no clear rules on selecting the final optimal profile model, multiple model fit indices such as absolute and relative model fit were used in the model selection process. In the profile naming process, covariates and distal outcomes were not added to the model. After profile naming, data on classification quality, such as logit values, were taken, which were used in a subsequent model to determine the variance of measurement error in the mixture model (Cho, 2019). The means of each class were manually fixed using logit values. Thus, the number of students assigned to classes and the ranking of classes were prevented from changing with the addition of covariates. Once the optimal number of hidden profiles was determined, covariates were theoretically included in the model to estimate their effect on profile membership.

Selecting the optimal number of classes is critical for meaningful interpretation of results (Tein et al., 2013). Latent Profile Analysis (LPA) employs four key criteria:

1 Information criteria

- AIC (Akaike Information Criterion): Lower values indicate better model fit.
- BIC (Bayesian Information Criterion): Favors parsimony; models with lowest values balance fit and complexity.
- Adjusted BIC: Enhances BIC’s performance in smaller samples, interpreted similarly.

2 Classification accuracy

- Entropy: Values ≥0.70 suggest acceptable profile separation, with ≥0.80 preferred (Celeux and Soromenho, 1996). Higher values (≥0.90) denote exceptional classification certainty.

3 Likelihood ratio tests

- LMRT: Significant *p*-values (*p* < 0.05) support k-profile models over (k-1)-profile alternatives (Lo et al., 2001).
- BLRT: More robust for nested model comparisons, with *p* < 0.001 indicating strong evidence for additional profiles.

4 Parsimony principle

- When consecutive models show diminishing fit improvements (e.g., <10% reductions in AIC/BIC between 3-vs. 4-profile solutions), simpler models are retained. This framework prioritizes substantive interpretability over marginal statistical gains, consistent with methodological guidelines for LPA implementation.

*In step 2*, we focused on estimating profile uncertainty rates before including covariates in the analysis. Using logit values from the Mplus output to classify uncertainty rates, information on classification quality was determined for future use in a model. As a result, the assignment logit probability values of each profile were calculated. This involved creating a variable for the most likely profile with measurement error rates. Each profile was identified based on logit values derived from the original mixture model results, ensuring that subsequent covariates and additional variables in the unconditional model did not influence profile membership.

*In Step 3*, covariates and distal outcomes are added to the model. In this step, the conditional means and variances of covariates and distal outcomes (loneliness, communication skills, happiness) are estimated for each latent class using a three-step latent class analysis in Mplus. The covariates part represents a regression analysis that examines how the latent class membership of the model is shaped under the influence of certain exogenous variables. This analysis reveals how the covariates in the model affect the probability of which latent class individuals belong to. Then, model restrictions are used to calculate differences between classes and omnibus tests are used to examine whether these differences are statistically significant.

## 4 Results

### 4.1 Latent phubbing profiles of university students

#### 4.1.1 Descriptive findings related to the factors, covariates and distal outcomes

Table 2 provides descriptive statistics related to the factors, covariates, and distal outcomes used for LPA in the study.

**Table 2:** Descriptive statistics related to the factors, covariates, and distal outcomes used in the study.

Variables and Categories	n (%)	M (SD)	Min	Max
Sub-factors of the phubbing scale				
Factor 1: Nomophobia		3.574 (1.652)	1	7
Factor 2: Interpersonal Conflict		2.093(1.307)	1	7
Factor 3: Self-Isolation		2.183 (1.408)	1	7
Factor 4: Problem Acknowledgment		2.779 (1.551)	1	7
Covariates				
Gender				
Female	497 (71.9)			
Male	194 (28.1)			
Age		22.50 (4.829)	17	57
Self-assessment as a smartphone addict				
I am not	194 (28.1)			
Partially	406 (58.8)			
I am addicted	91 (13.2)			
Family’s economic situation				
Low	142 (20.5)			
Medium	529 (76.6)			
High	20 (2.9)			
Number of Friends				
Less than 5	195 (28.2)			
6–15	331 (47.9)			
16–50	115 (16.6)			
More than 50	50 (7.2)			
Weekly Frequency of Meeting with Friends				
0–3 Days	480 (69.5)			
4–5 Days	137 (19.8)			
6–7 Days	74 (10.7)			
Feeling depressed				
Yes	317 (45.9)			
No	374 (54.1)			
Sleep deprivation due to social media or phone use				
Yes	310 (44.9)			
No	381 (55.1)			
Distal Outcomes				
Loneliness		2.316 (0.863)	1	5
Communication		3.724 (0.876)	1	5
Happiness		3.108 (0.736)	1	5

Table 2 presents the descriptive statistics for the factors, covariates, and distal outcomes examined in this study. The mean scores for the sub-factors of the phubbing scale reveal moderate levels of phubbing behaviors among participants, with “Nomophobia” (M = 3.574, SD = 1.652) being the most prominent factor, followed by “Problem Acknowledgment” (M = 2.779, SD = 1.551). In contrast, “Interpersonal Conflict” (M = 2.093, SD = 1.307) and “Self-Isolation” (M = 2.183, SD = 1.408) were reported at slightly lower levels.

Regarding covariates, the sample consisted primarily of female participants (71.9%). The average age was 22.50 years (SD = 4.829), ranging from 17 to 57 years. A majority of participants identified themselves as only partially addicted to smartphones (58.8%), and the family’s economic situation was predominantly reported as “Medium” (76.6%). Most participants reported having 6 to 15 friends (47.9%) and meeting friends 0–3 days per week (69.5%). Nearly half of the participants reported feeling depressed (45.9%) and experiencing sleep deprivation due to social media or phone use (44.9%).

In terms of distal outcomes, the mean scores suggest moderate levels of loneliness (M = 2.316, SD = 0.863) and happiness (M = 3.108, SD = 0.736), with relatively higher self-reported communication skills (M = 3.724, SD = 0.876).

#### 4.1.2 Latent phubbing profiles

Table 3 presents the Latent Profile Analysis (LPA) fit indices for models with varying profiles. The Akaike Information Criterion (AIC), Bayesian Information Criterion (BIC), and Adjusted BIC values consistently decrease with the addition of more profiles, suggesting improved model fit up to a certain point.

**Table 3:** LPA fit indices.

Number of profiles	AIC	BIC	Adjusted BIC	Entropy	LMRT|BLRT *p* value
1	7.855.889	7.892.194	7.866.793		–
2	6.551.084	6.610.080	6.568.803	0.958	0.001|0.001
3	6.084.011	6.165.698	6.108.545	**0.903**	**0.001|0.001**
4	5.938.812	6.043.189	5.970.160	0.915	0.001|0.001
5	5.803.232	5.930.300	5.841.395	0.923	0.061|0.001
6	5.690.238	5.839.997	5.735.217	0.882	0.134|0.001

AIC, Akaike Information Criterion; BIC, Bayesian Information Criterion; Adjusted BIC, Sample-size Adjusted BIC; Entropy, Classification Entropy; LMRT, Lo-Mendell-Rubin Adjusted Likelihood Ratio Test; BLRT Bootstrap Likelihood Ratio Test.

Based on the fit indices presented in Table 3, the three-profile model was determined to be the most appropriate solution for this study. While both the three-profile and four-profile models show a reduction in Akaike Information Criterion (AIC) and Bayesian Information Criterion (BIC) values compared to the two-profile model, the decrease between the three-profile and four-profile models is not substantial. This suggests that the additional complexity of a four-profile model does not significantly enhance model fit over the three-profile model (Tein et al., 2013). Furthermore, the entropy value for the three-profile model is 0.903, indicating a high level of classification accuracy and precise separation between the profiles. High entropy values (closer to 1) suggest that the model effectively distinguishes between the latent profiles, which is crucial for the interpretability and reliability of the results.

Considering these factors—the lack of significant improvement in AIC and BIC values beyond three profiles, the high entropy value for the three-profile model, and the non-significant LMRT *p*-values for models with more than three profiles—the three-profile model is considered the most parsimonious and effective solution. Focusing on the critical characteristics and factors of each of the three profiles, we can describe them as follows:

Profile 1: Low Phubbing Risk (465 students; 67.294%) Most prominent feature: Lack of Problem Acknowledgement (Problem Acknowledgment: −0.534) Students in this group had the lowest risk of phubbing compared to the other two groups. They were least concerned about the lack of problem acknowledgement. They had limited concerns about Nomophobia (−0.462), Interpersonal Conflict (−0.479) and Self-Isolation (−0.461) related to social negligence toward the physical environment during phone use.

Profile 2: Moderate Phubbing Risk (166 students; 24.023%) The most prominent feature is Problem Acknowledgement (Problem Acknowledgement: 0.756). Compared to the other two groups, Students in this group have a moderate risk of phubbing. Their biggest concern is accepting the problem. They also have moderate concerns about Nomophobia (0.741), Interpersonal Conflict (0.530), and Self-Isolation (0.365).

Profile 3: High Phubbing Risk (60 students; 8.683%) Most prominent feature: Isolation (Self-Isolation: 2.511). Students in this group have the highest risk of phubbing compared to the other two groups. Their biggest concern is isolation. They also have deep concerns about interpersonal conflict (2.188), problem acknowledgement (1.970), and nomophobia (1.459).

**Figure 1:**
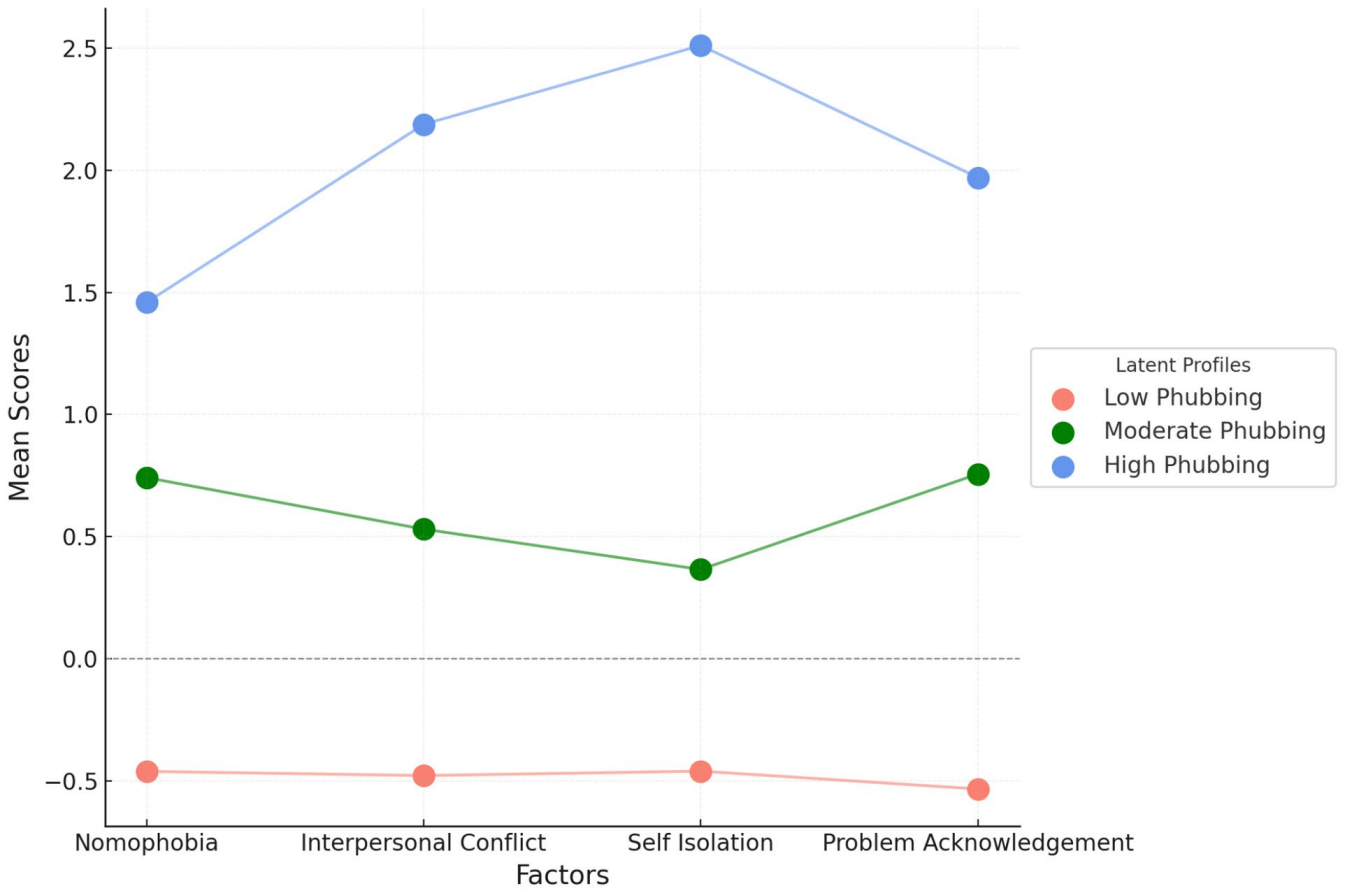
Profile plot of latent profiles.

Figure 1 shows the mean scores between individuals with different risk levels of phubbing behavior. The graph compares three groups with low, moderate, and high phubbing risk.

### 4.2 The effect of covariates on students’ phubbing profiles

Table 4 shows the multinomial logistic regression analysis to determine how the covariates used in the study (gender, age, smartphone addiction, insomnia, depression, number of friends, frequency of meeting friends and socio-economic status) affect the profiles.

**Table 4:** Logistic regression results.

Covariates	Moderate (OR)	S.E.	z	*p*	High (OR)	S.E.	z	*p*
Female (ref: Male)	1.706	0.154	−3.033	0.002**	1.473	0.159	−3.866	<0.001**
Age	1.007	0.027	0.241	0.809	1.022	0.045	0.491	0.624
Depression: Yes (ref: No)	0.503	0.189	−1.646	0.100	0.487	0.244	−1.154	0.248
Sleep deprivation: Yes (ref: No)	0.648	0.111	−5.089	<0.001**	0.737	0.110	−6.311	<0.001**
Classes of students								
Class_1	1.357	0.434	0.702	0.482	0.948	0.432	−0.125	0.901
Class_2	1.047	0.803	1.301	0.193	1.258	0.678	0.339	0.734
Class_3	1.891	1.373	0.464	0.643	1.194	1.377	0.129	0.898
Class_4 (Reference)	–	–	–	–	–	–	–	–
Self-assessment as a smartphone addict								
I am not	0.051	0.027	−35.383	<0.001**	0.037	0.025	−38.439	<0.001**
Partially	0.339	0.116	−5.722	<0.001**	0.157	0.060	−14.163	<0.001**
I am addicted (Reference)	–	–	–	–	–	–	–	–
Family’s economic situation								
Low	5.516	1.238	0.572	0.567	1.665	0.451	−1.088	0.277
Medium	4.883	1.068	0.549	0.583	1.807	0.472	−0.864	0.387
High (Reference)	–	–	–	–	–	–	–	–
Number of Friends								
Less than 5	4.343	0.866	0.540	0.589	1.786	0.399	−1.053	0.293
6–15	4.352	0.820	0.573	0.567	1.833	0.373	−1.057	0.290
16–50	4.161	0.849	0.502	0.616	2.036	0.469	−0.616	0.538
More than 50 (ref.)	–	–	–	–	–	–	–	–
Weekly Frequency of Meeting with Friends								
0–3 Days	5.290	0.740	0.900	0.368	2.875	0.576	0.098	0.922
4–5 Days	5.384	0.858	0.796	0.426	4.457	0.911	0.544	0.587
6–7 Days (Reference)	–	–	–	–	–	–	–	–

*p < 0.05, **p < 0.01, ***p < 0.001. OR, Odds Ratio.

According to Table 4, being a female in the Moderate Phubbing profile significantly increases the likelihood of carrying the risk of moderate phubbing compared to men (OR = 1.706, *p* = 0.002). This indicates that females are approximately 1.7 times more likely to be at moderate phubbing risk than males. Again, in the High Phubbing Risk profile, being female significantly increases the likelihood of having a high phubbing risk compared to males (OR = 1.473, *p* < 0.001). This indicates that females are approximately 1.5 times more likely to be at high phubbing risk than males.

In our study, the variables of age, depression, grades of students, economic status of the family, number of friends and frequency of meeting with friends did not have a significant effect on moderate and high phubbing risk profiles.

Table 4 shows that sleep deprivation significantly reduced the probability of being in the moderate phubbing risk category by 35.20% (OR = 0.648, *p* < 0.001). This indicates that sleep-deprived individuals are less likely to carry phubbing risk. Furthermore, sleep deprivation significantly reduced the odds of being in the high phubbing risk category by 26.30% (OR = 0.737, *p* < 0.001). This indicates that individuals who are sleep-deprived are less likely to be at risk of phubbing.

According to the participants’ self-assessment of themselves as smartphone addicts, concerning those who said they were addicted, those who said they were not addicted were significantly 94.90% [(1–0.051)*100] less likely to be in the Moderate Phubbing Risk profile (OR = 0.051, *p* < 0.001). Similarly, the probability of being in the High Phubbing Risk profile was 96.30% (OR = 0.037, *p* < 0.001). Concerning those who rated themselves as smartphone-addicted, those who rated themselves as somewhat addicted were significantly 66.10% less likely to be in the Moderate Phubbing Risk profile (OR = 0.339, *p* < 0.001). Similarly, they were 84.30% less likely to be in the High Phubbing Risk profile (OR = 0.157, *p* < 0.001).

### 4.3 The relationship between distal outcomes and phubbing profiles of university students

**Figure 2:**
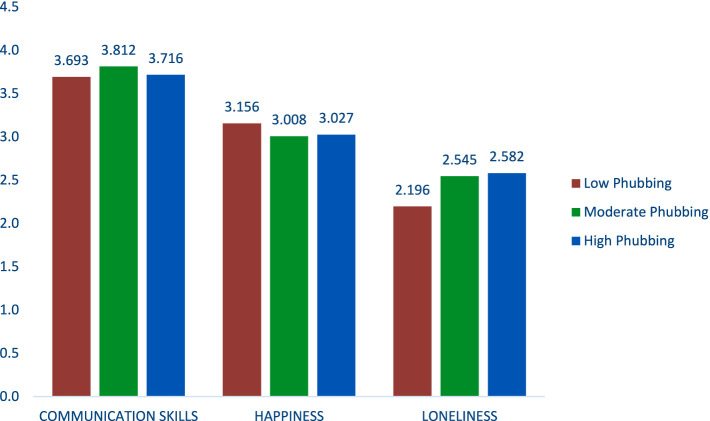
Profile mean comparison in point of distal outcomes.

Figure 2 depicts the mean scores of distal outcomes—loneliness, communication skills, and happiness—across three phubbing profiles (High, Low, and Moderate Phubbing).

Figure 2 illustrates that the differences between the three phubbing profiles are minimal for communication skills and happiness, with all profiles showing relatively similar scores. However, a noticeable difference emerges in loneliness levels, where the Low Phubbing group exhibits significantly lower loneliness levels than the Moderate and High Phubbing groups. By examining the profile mean differences, we can explore how phubbing behaviors are associated with emotional and social well-being among students.

Table 5 presents the results, highlighting the significant and non-significant differences between the profiles regarding loneliness, communication skills, and happiness.

**Table 5:** Differences among profiles.

Distal outcomes	Difference	S.E.	z	*p*	Sig. and direction
Loneliness					
Low vs. Moderate	−0.349	0.111	−3.150	0.002**	Sig. (Low< Moderate)
Low vs. High	−0.387	0.140	−2.755	0.006**	Sig. (Low < High)
Moderate vs. High	−0.038	0.116	−0.230	0.818	Not Significant
Communication Skills					
Low vs. Moderate	−0.119	0.103	−1.156	0.248	Not Significant
Low vs. High	−0.023	0.133	−0.177	0.860	Not Significant
Moderate vs. High	0.095	0.154	0.620	0.535	Not Significant
Happiness					
Low vs. Moderate	0.148	0.098	1.512	0.131	Not Significant
Low vs. High	0.129	0.101	1.287	0.198	Not Significant
Moderate vs. High	−0.019	0.125	−0.149	0.882	Not Significant

Table 5 illustrates the differences in loneliness, communication skills, and happiness across the three phubbing profiles. The most significant findings relate to loneliness, where the Low profile reported significantly lower levels than the Moderate (*p* = 0.002) and High (*p* = 0.006) profiles. However, no significant difference between the Moderate and High profiles regarding loneliness was observed.

No significant differences were found among the three profiles for communication skills. Similarly, no significant differences emerged for happiness across the profiles, with all comparisons yielding non-significant results. In conclusion, the critical distinction across phubbing profiles lies in their relationship with loneliness, while communication skills and happiness do not significantly vary. These findings suggest that loneliness is the most affected outcome among individuals displaying higher phubbing behaviors.

## 5 Discussion

In this study, we used LPA to identify various phubbing profiles among university students and explore the connection between these profiles and distal outcomes like loneliness, communication skills, and happiness. The study also investigated the influence of some factors, such as gender, age, smartphone addiction, sleep deprivation, depression, number of friends, frequency of meeting friends and socioeconomic status.

The LPA yielded three phubbing profiles: Low, Moderate, and High Phubbing Risk. The most extensive profile in the sample was the Low Phubbing Risk (67.3%), which refers to most students having lower levels of problematic phone use, unlike what was reported in the previous studies that documented the prevalence of phubbing among university students. The reasons for these incongruences can be found in cross-cultural and contextual differences and the samples’ different characteristics. On the other hand, identifying the Moderate (24%) and the High Phubbing Risk (8.7%) groups indicates that many students experience moderate to high phubbing, which can have considerable social and psychological consequences.

The results showed that gender predicted phubbing (female students having a higher probability of exhibiting phubbing behavior compared with male students), which is consistent with previous research about excessive smartphone use where females tend to use smartphones excessively for the maintenance of social relationships and engagement with social media (Bitar et al., 2022; Cebollero-Salinas et al., 2022). Concerning age, it did not act as a differentiating factor of phubbing, which goes against previous studies that reported an association with higher phubbing rates with younger age groups, such as between 18 and 29 years old (Maftei and Măirean, 2023). This implies that in a university context immersed in digital culture and highly engaged with smartphone use across all age groups, phubbing might more strongly correlate to the widespread use of smartphones rather than the age difference.

It turned out that heavy smartphone usage significantly predicted phubbing, which aligns with the observation of other researchers indicating smartphone addiction to be the most critical factor in propelling phubbing behaviors. Kwon et al. (2013) and Ivanova et al. (2020) have all shown that the stronger the addiction, the more likely you are to indulge in phubbing on a computer or smartphone. Students who are highly dependent on their smartphones will prioritize digital over face-to-face interactions due to fear that others will take their phones away, FOMO, and the need to check multiple sites to compare themselves. They are much more likely to interrupt or ignore those right before them than those less obsessed with their smartphones. Our research significantly shows the need to try to curb the predicament of smartphone addiction if we want to decrease the rates of phubbing behaviors.

It is, therefore, somewhat surprising that lack of sleep did not produce the expected results. People with a sleeping problem were less likely to phub. Such a finding goes against research that shows night-time smartphone use, known to decrease sleep quality (Tamura et al., 2017), can worsen the damaging effects of sleep deprivation. An explanation might be that those who are sleep-deprived might have less energy or cognitive resources to engage in social activities (including phubbing) in the daytime.

More broadly, the research identified the heaviness of students’ phubbing behavior and the relationships with socioeconomic status, number of friends, and frequency of contact between one individual and other students. It is observed that socioeconomic status has a complex relationship with phubbing. Results derived from the above studies (Chu et al., 2021; Chu et al., 2023) indicated that socioeconomic status impacts phubbing by social anxiety and family relationships. That indicates socioeconomic status, a family background that appears to have a comprehensive effect on an individual’s social and emotional attitude rather than simply being a factor. The surveys did not find a direct impact of socioeconomic status with phubbing, so Evans and Kantrowitz (2002) have emphasized the complexity of socioeconomic status and its play in health. The consequence of socioeconomic status is not as simple as the relationship between the parent’s educational status and money on their children’s socializing and emotional attitudinal sharing. Additional factors such as individual-independent factors, family background, society habits, etc., have all impacted how one reacts under a diagnosis of a similar socioeconomic status.

Another factor taken into consideration was the frequency of friends and meetings. Previous evidence has highlighted that the quantity and frequency of social interactions determine phubbing behaviors (Parus et al., 2021; Uncu et al., 2024). However, the findings showed that the frequency of friends and meetings had not influenced phubbing behaviors. The results indicate that phubbing behaviors have been determined by individual traits rather than the number of social interactions (Safdar Bajwa et al., 2023; Kwon et al., 2013).

In this study, depression did not affect phubbing behaviors. Previous studies have reported a significant association between depression and phubbing. For example, two recent studies reported higher phubbing behaviors in people with higher levels of depression and anxiety, Ivanova et al. (2020) and García-Castro et al. (2022). However, this time, the prominent increase in college students’ phubbing behaviors resulted not from mental health factors, such as depression, but from users’ connective habits, such as digital addiction and social media use. McDaniel and Radesky (2017) explained that coping with mental health issues such as depression might take various forms, and phubbing might not always be the go-to pattern.

Regarding the long-term results, students with a higher risk for phubbing reported feeling significantly lonelier. This finding echoes what is reported in previous studies on phubbing and social isolation (Maftei and Măirean, 2023; Sun and Wong, 2024). Since phubbing is based on replacing face-to-face interaction with interaction through technological devices, it is natural that they become less socially involved and feel rejected within their conversational partner and the social group. We did not find any differences between the profiles and their communication skills or happiness, which suggested that in a university context, phubbing directly affects these results. This finding contradicts previous research on phubbing that has established links between phubbing and lower communication quality and relationship satisfaction (Chotpitayasunondh and Douglas, 2018; Ayar and Gürkan, 2021). It is possible that even if university students are phub, they still maintain good communication skills and that other factors, such as university achievements or social media engagement, can influence their level of happiness rather than phubbing itself.

The current research contributes to the growing body of literature on phubbing by systematically investigating the prevalence and correlates of phubbing among emerging adults, particularly college students. This research sheds light on the patterns of phubbing practices in a college setting by elucidating distinct phubbing profiles and the associations between different profiles, demographic characteristics, and psychological outcomes. Also, this study highlights the crucial role of smartphone addiction as a driving force of phubbing behavior, underscoring the need for anti-phubbing interventions to attend to digital addiction. Furthermore, the current study’s finding that loneliness is a critical outcome of phubbing behavior aligns with previous research and further highlights the need to continue exploring the bidirectional relation between these two variables.

For future research, it would be interesting to use longitudinal designs to explore whether the presence and frequency of phubbing over time can predict long-term changes in students’ social and psychological well-being. Furthermore, investigating the relative effects of contextual factors on the associations between phubbing, communication abilities, and happiness would provide a more nuanced understanding of the heterogeneity of these outcomes in different environments. Given the dogmatic attention that digital transactions receive in educational and professional contexts, further research on preventing the adverse effects of phubbing and developing efficient remedies would be welcome. Similarly, intervention studies targeting smartphone addiction and developing healthier digital behaviors in university students would be worth creating and testing.

The research outlined above warrants consideration within the context of certain limitations. The study’s cross-sectional design precludes definitive conclusions about potential links between phubbing, covariates, and distal outcomes. Additionally, as the study solely focused on students within a single university context, the generalizability of the results to student samples from diverse cultural backgrounds may be limited.

## 6 Conclusion

This study identified distinct phubbing profiles among university students using latent profile analysis (LPA) and examined predictors and consequences associated with these profiles. The analysis revealed three meaningful phubbing profiles: “Low risk,” “Moderate risk,” and “High risk,” with the majority of students classified in the “Low risk” group, indicating that most students were not highly problematic smartphone users. Nonetheless, the presence of “Moderate” and “High risk” groups highlights that problematic phone use remains an important concern for certain student segments.

In addressing the second research question regarding predictors, gender and smartphone addiction emerged as significant factors influencing students’ phubbing profiles. Females were more likely to exhibit higher phubbing risk profiles compared to males, aligning with existing literature on gender differences in smartphone usage behaviors. Smartphone addiction, notably, was identified as the strongest predictor of phubbing behavior, underscoring the critical role digital dependency plays in fostering such behavior. Other examined factors, including insomnia, depression, socioeconomic status, number of friends, and frequency of social interactions, did not significantly predict phubbing profiles.

Regarding the third research question about distal outcomes, increased phubbing risk was significantly associated with higher levels of loneliness, supporting existing evidence that excessive reliance on device-mediated social interactions can lead to greater social isolation. However, no significant differences were found between phubbing profiles in terms of communication skills and happiness, suggesting these outcomes may be less directly impacted among university students.

This study contributes to the growing body of research on phubbing, providing a more systematic account of its prevalence, correlates, and consequences among university students. It emphasizes the importance of addressing smartphone addiction as a central driver of phubbing and provides evidence that excessive smartphone involvement, including phubbing, may promote loneliness. Future research may consider longitudinal designs to trace the long-term effects of phubbing on social and psychological well-being, as well as intervention studies aimed at fostering healthy digital lifestyles and preventing the adverse consequences of phubbing.

## Data Availability

The datasets presented in this study can be found in online repositories. The names of the repository/repositories and accession number(s) can be found at: https://figshare.com/s/02e46fe25ae094542b93.
